# Combining radiomic features with a miRNA classifier may improve prediction of malignant pathology for pancreatic intraductal papillary mucinous neoplasms

**DOI:** 10.18632/oncotarget.11768

**Published:** 2016-08-31

**Authors:** Jennifer B. Permuth, Jung Choi, Yoganand Balarunathan, Jongphil Kim, Dung-Tsa Chen, Lu Chen, Sonia Orcutt, Matthew P. Doepker, Kenneth Gage, Geoffrey Zhang, Kujtim Latifi, Sarah Hoffe, Kun Jiang, Domenico Coppola, Barbara A. Centeno, Anthony Magliocco, Qian Li, Jose Trevino, Nipun Merchant, Robert Gillies, Mokenge Malafa

**Affiliations:** ^1^ Cancer Epidemiology, Moffitt Cancer Center and Research Institute, Tampa, Florida, USA; ^2^ Gastrointestinal Oncology, Moffitt Cancer Center and Research Institute, Tampa, Florida, USA; ^3^ Diagnostic Imaging and Interventional Radiology, Moffitt Cancer Center and Research Institute, Tampa, Florida, USA; ^4^ Cancer Imaging and Metabolism, Moffitt Cancer Center and Research Institute, Tampa, Florida, USA; ^5^ Biostatistics and Bioinformatics, Moffitt Cancer Center and Research Institute, Tampa, Florida, USA; ^6^ Radiation Oncology, Moffitt Cancer Center and Research Institute, Tampa, Florida, USA; ^7^ Anatomic Pathology, Moffitt Cancer Center and Research Institute, Tampa, Florida, USA; ^8^ Department of Radiology, Tianjin Medical University Cancer Institute and Hospital, Tianjin, China; ^9^ Department of Surgery, Division of General Surgery, University of Florida Health Sciences Center, Gainesville, Florida, USA; ^10^ Department of Surgery, Sylvester Comprehensive Cancer Center at the University of Miami Miller School of Medicine, Miami, Florida, USA; ^11^ Department of Clinical Surgery/Surgical Oncology, Palmetto Health/USC School of Medicine, Columbia, South Carolina, USA

**Keywords:** radiomics, miRNA, risk stratification, pre-malignant lesions, pancreas

## Abstract

Intraductal papillary mucinous neoplasms (IPMNs) are pancreatic cancer precursors incidentally discovered by cross-sectional imaging. Consensus guidelines for IPMN management rely on standard radiologic features to predict pathology, but they lack accuracy. Using a retrospective cohort of 38 surgically-resected, pathologically-confirmed IPMNs (20 benign; 18 malignant) with preoperative computed tomography (CT) images and matched plasma-based ‘miRNA genomic classifier (MGC)’ data, we determined whether quantitative ‘radiomic’ CT features (+/- the MGC) can more accurately predict IPMN pathology than standard radiologic features ‘high-risk’ or ‘worrisome’ for malignancy. Logistic regression, principal component analyses, and cross-validation were used to examine associations. Sensitivity, specificity, positive and negative predictive value (PPV, NPV) were estimated. The MGC, ‘high-risk,’ and ‘worrisome’ radiologic features had area under the receiver operating characteristic curve (AUC) values of 0.83, 0.84, and 0.54, respectively. Fourteen radiomic features differentiated malignant from benign IPMNs (p<0.05) and collectively had an AUC=0.77. Combining radiomic features with the MGC revealed an AUC=0.92 and superior sensitivity (83%), specificity (89%), PPV (88%), and NPV (85%) than other models. Evaluation of uncertainty by 10-fold cross-validation retained an AUC>0.80 (0.87 (95% CI:0.84-0.89)). This proof-of-concept study suggests a noninvasive radiogenomic approach may more accurately predict IPMN pathology than ‘worrisome’ radiologic features considered in consensus guidelines.

## INTRODUCTION

To revolutionize the early detection of cancer, there is a need to replace invasive and risky tissue biopsies not representative of the entire tumor with noninvasive tests reflecting the tumor and its environment. Such a discovery is sorely needed for pancreatic ductal adenocarcinoma (PC), the deadliest of the leading causes of cancer death in the United States, with a five-year survival rate of only 8% [1, 2]. PC is currently the third leading cause of cancer death, and is projected to become the second leading cause around 2020. Most cases (85%) present with metastases because of the lack of accurate methods to detect disease at an early, operable stage [1]. The detection and treatment of precursor lesions offers great promise for reducing morbidity and mortality.

Intraductal papillary mucinous neoplasms (IPMNs) are PC precursors accounting for nearly half of the ∼150,000 asymptomatic pancreatic cysts detected incidentally in up to 2.6% of computed tomography (CT) scans and 19.9% of magnetic resonance imaging (MRI) studies each year [3]. IPMNs are challenging to manage due to the inability to predict which lesions can be safely monitored, which may progress to invasion, and which have associated invasion [3]. The only way to treat IPMNs and examine severity (which ranges from low- and moderate-grade dysplasia to high-grade dysplasia and invasive carcinoma) is through surgical resection and pathological evaluation. However, pancreatic resection is associated with an operative mortality of 2-4% and morbidity of 40-50% [4]. One clue regarding histologic severity can be obtained radiologically by investigating whether the IPMN(s) present within the main pancreatic duct (MD-IPMN), side branch ducts (BD-IPMN), or both (mixed-IPMN); surgical series confirm that IPMNs with MD involvement harbor a higher risk of malignancy (∼60%, range: 11-81%) and more rapid growth compared to BD-IPMNs (26%, range: 6-47%) [5]. Consensus guidelines for IPMN management known as ‘Sendai guidelines’ exist [5] and rely on standard radiographic and clinical features. These guidelines [5] suggest that patients with ‘high risk stigmata’ (MD involvement/ dilatation > 10 mm, obstructive jaundice with a cystic lesion in the pancreatic head, or an enhanced solid component/nodule within the cyst) undergo resection, as most harbor high-grade or invasive disease [5]. On the other hand, it is recommended that presumed BD-IPMNs with ‘worrisome features’ (MD dilation 5-9 mm, cyst size > 3 cm, thickened enhanced cyst walls, non-enhanced mural nodules, or acute pancreatitis) undergo surveillance with an invasive endoscopic ultrasound-guided fine needle aspirate (EUS-FNA) procedure despite poor sensitivity and technical complications [3, 6]. Although the consensus guidelines provide a valuable framework for management, the agreement between the preoperative diagnosis and pathologic examination is inaccurate in a substantial proportion (30-70%) of cases [5, 7–11]. Thus, novel markers of malignant pathology are needed, especially for cases that do not appear to present with high-risk stigmata.

miRNAs are excellent candidate biomarkers of pancreatic tumorigenesis because of their tissue-specific expression, stability in biofluids, and their ability to regulate hundreds of genes and biological pathways [12]. We recently conducted genome-wide miRNA analysis using tissue [13] and blood plasma [14] from a cohort of 42 patients with surgically-resected, pathologically-confirmed IPMNs. Our unbiased analysis of 800 miRNAs from archived plasma using Nanostring's nCounter digital technology™ [14] revealed a 5-miRNA genomic classifier (MGC) that included miR-200a-3p, miR-1185-5p, miR-33a-5p, miR-574-4p, and miR-664b and discriminated between 21 malignant (classified as high-grade or invasive) and 21 benign IPMNs (classified as low- or moderate-grade) (p = 0.005, area under the receiver operating characteristic curve (AUC) = 0.73 (95% CI:0.58-0.89). These miRNAs had 2-3 fold lower expression in malignant compared to benign cases, supporting a tumor suppressor role. Recent studies of other cancers [15, 16] suggest the ability to predict lesion severity may improve further by combining genomic data with quantitative radiologic features.

Radiomics refers to the high-throughput extraction and analysis of quantitative features from standard-of-care medical images with the intent of generating mineable databases that can be used to build predictive models relating imaging features (or ‘radiophenotypes’) to clinical outcomes [17]. Categories of radiomic features such as tumor signal intensity, shape characterization, and texture have the following advantages over and/or provide enhancements to standard radiologic features [18–24]: they i.) represent quantitative, objective measures, ii.) reflect tumor heterogeneity and sub-regional habitats, iii.) can be more strongly linked to clinical outcomes, iv.) can be more reproducible and stable, and v.) can improve diagnostic accuracy when combined with standard radiologic features. We hypothesized that adopting a radiomic approach could enhance preoperative prediction of IPMN malignancy (either alone or in combination with the MGC) by uncovering diagnostic and biologic information ‘hidden’ in routinely acquired images. CTs are the most widely used imaging modality in oncology and have emerged as a preferred modality for the detection and characterization of pancreatic cysts because of its widespread availability, high spatial and temporal resolution, short scanning duration, high-quality multiplanar image display [25], and similar accuracy as MRIs for characterizing pancreatic cysts as benign or malignant [26]. Radiomics evaluations of pre-treatment CT scans have been conducted by our team [19, 21, 22, 24, 27] and others [18, 28–32], with associations reported between radiophenotypes and clinical outcomes. Moreover, ‘radiogenomics’ approaches have been used to link imaging features to underlying genomic information [15, 33–39]. The goal of this study was to determine whether radiomic features extracted from preoperative CT scans, either alone or with miRNA data, may improve prediction of IPMN pathology beyond that provided by standard radiologic or clinical features encompassed by current consensus guidelines [5].

## RESULTS

### Study population characteristics

Selected clinical, epidemiologic, and imaging characteristics of the 38 cases (20 benign; 18 malignant) having matched pre-operative CT and MGC data are in Table 1. Seventy-two percent with malignant pathology had MD involvement on CT vs. 20% with benign pathology (p = 0.003). Mean cyst size was lower in the benign compared to the malignant group (2.8 vs. 3.9 cm), p = 0.018. Consistent with published data (8, 9, 48), most cases (83%) with malignant pathology had ≥1 “high risk stigmata” (MD involvement/ dilatation > 10 mm, obstructive jaundice with a cystic lesion in the pancreatic head, or an enhanced solid component within the cyst), vs. 15% of those with benign pathology (p < 0.001). Having one or more “worrisome” features (ie. MD dilation 5-9 mm, cyst size > 3 cm, thickened enhanced cyst walls, non-enhanced mural nodules, or acute pancreatitis) was not associated with malignancy (p = 0.73), suggesting surgery may not have been indicated. PC1 of the MGC was significantly lower in the malignant group compared to the benign group (p < 0.001).

**Table 1:** Characteristics of IPMN patients with pre-operative CTs and miRNA data (N=38)

Variable	Benign^1^ IPMNs (n=20)	Malignant^2^ IPMNs (n=18)	*P*-value
**Age at diagnosis, mean (SD)(yrs)**	68.0 (10.4)	70.9 (11.7)	0.422
**Gender**			
**Male**	5 (25)	8 (44)	0.096
**Female**	15 (75)	10 (56)	
**Race**			
**White, Non-Hispanic**	20 (100)	16 (89)	0.218
**Black**	0 (0)	2 (11)	
**Jaundice as presenting symptom** **Yes** **No** **Pre-operative serum CA 19-9 levels, mean (SD)(ng/mL)** **Pre-operative serum albumin levels, mean (SD)(ng/mL)** **Predominant tumor location**	1 (5) 19 (95) 165 (330) 4.4 (1.0)	5 (28) 13 (72) 88.9 (305) 3.9 (0.6)	0.083 0.300 **0.012**
**Pancreatic Head**	5 (25)	8 (13)	**0.023**
**Pancreatic Body or Tail** **Diffuse**	15 (75) 0 (0)	5 (53) 2 (13)	
**Type of ductal communication** **Main duct or mixed** **Branch duct** **Main duct dilatation**	4 (20) 16 (80)	13 (72) 5 (28)	**0.003**
**Diffuse** **Segmental** **None**	1 (5) 3 (15) 16 (80)	8 (50) 5 (31) 3 (19)	**<0.001**
**Size of largest cyst, mean (range) (cm)** **Solid component or mural nodule** **Yes** **No**	2.8 (1.1-6.6) 3 (15) 17 (85)	3.9 (1.6-5.4) 9 (50) 9 (50)	**0.018** **0.035**
**High risk stigmata** **Yes** **No** **Worrisome features** **Yes** **No** **5 miRNA genomic classifier (MGC), mean (SD) PC1 expression**	3 (15) 17 (85) 13 (65) 7 (35) 0.7	15 (83) 3 (17) 13 (72) 5 (28) -0.7	**<0.001** 0.734 **<0.001**

Data represent counts (percentages) unless otherwise indicated. Counts may not add up to the total due to missing values, and percentages may not equal 100 due to rounding.

^1^ Benign IPMNs are represented by 4 low-grade and 16 moderate-grade IPMNs.

^2^ Malignant IPMNs are represented by 11 high-grade and 7 invasive IPMNs.

### Analysis of standard radiologic and clinical characteristics and miRNA data

**Figure 1:**
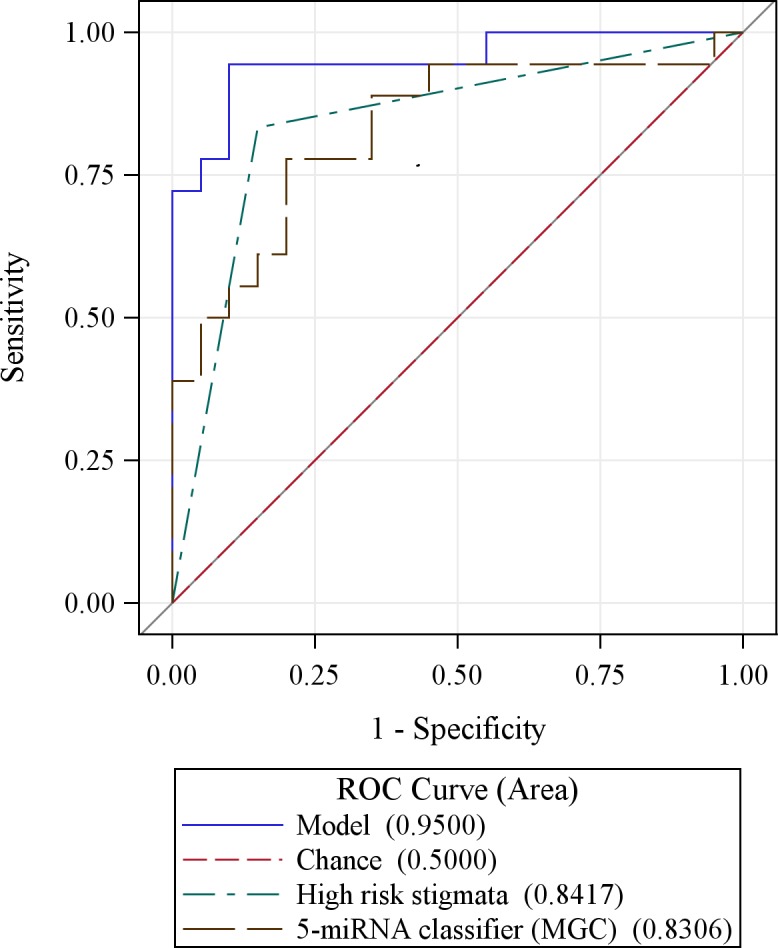
A model that combines the 5 miRNA genomic classifier signature (MGC) with high risk stigmata is more accurate in predicting IPMN malignancy than either variable alone

Using variables from Table 1, multiple logistic regression analyses revealed that only high risk stigmata and the MGC retained significance (OR (95% CI): 43.0 (4.64-398), p = 0.001 and OR (95% CI): 0.30 (0.10-0.86), p = 0.026, respectively). The AUC value was 0.95 for the model with both variables, compared to 0.84 and 0.83 for high risk stigmata (p = 0.063) and the MGC (p = 0.038) individually (Figure 1).

Since medical management is less clear for the estimated 60% of presumed BD-IPMN cases who have worrisome features (and do not present with high risk stigmata) (5), it was most important that we assess the added value of the MGC in that subset of patients. While worrisome features alone could not predict malignant pathology much better than chance (AUC = 0.54), the AUC increased to 0.83 when incorporating the MGC, primarily due to increased specificity. For example, when restricting to the 20 patients who did not present with high-risk stigmata, the specificity of the MGC and worrisome features were 70.6% and 35.3%, respectively. Finally, a model that solely considered demographic and clinical predictors of IPMN pathology highlighted previously (49-53) (age at diagnosis, gender, presence of symptoms) had an AUC (95%CI) = 0.73 (0.56-0.89).

### Analysis of radiomic data

**Figure 2:**
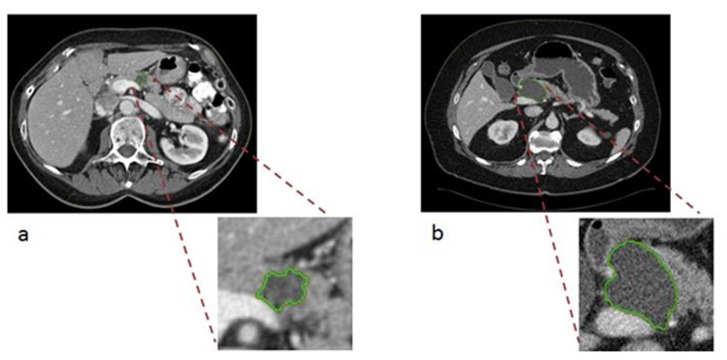
Semi-automated segmentation of two IPMN patient CT scans at the selected central slice **a.** Axial venous phase images through the abdomen demonstrate a cystic mass in the pancreatic head/neck measuring up to 3.5 cm. This lesion contains a non-enhancing soft tissue mural nodule (arrow). **b.** Axial venous phase images through the abdomen demonstrate an ovoid, homogeneous appearing cystic mass measuring up to 4.8 cm in greatest dimension. No internal enhancing soft tissue nodules were seen within the lesion.

Radiomic features were successfully extracted for 37 of the 38 cases; features could not be extracted for one benign case from an outside hospital because only digitized film was available and there were no coronal views. Univariate analysis of the 112 radiomic CT features revealed 14 features (11 textural, including histogram, wavelet, laws, and co-occurrence/run-length, and 3 non-textural, all size &shape) that differentiated malignant from benign IPMNs (p < 0.05) (Table 2). The most statistically significant features were textural and included histogram entropy layer 1 (OR (95% CI): 3.77 (1.34-10.63), p = 0.012) and run-length features G1 D0 LGRE Layer 1 (OR (95% CI): 4.30 (1.37-13.49), p = 0.013). Statistically significant non-textural features that were associated with an increased likelihood of malignant pathology included border length and width, whereas radius of the largest enclosed ellipse was associated with a decreased likelihood of malignant pathology (Table 2). Collectively, the 14 radiomic features (defined as ‘Features PC1’) had a diagnostic accuracy higher than worrisome features (AUC = 0.77 versus 0.54). ‘Features PC1’ explained 61% of the variability in the data, suggesting it represents the 14 most promising radiomic features well. Of clinical importance, there were three cases for whom the final pathology was benign that had worrisome features on preoperative imaging yet were correctly classified as true negatives (benign) via radiomics using the Features PC1 score; thus radiomic features may have helped to avoid overtreatment with surgery. An image from one of these cases is displayed in Figure 2a. On the other hand, there was one case for whom the final lesion pathology was malignant but there were no high risk stigmata on preoperative imaging (only one worrisome feature of cyst size > 3 cm) and radiomics classified the case as a true positive (malignant); thus radiomics may have aided in directing management towards a necessary surgery to remove what turned out to be a high-grade lesion (Figure 2b).

**Table 2:** Pre-operative radiomic CT features associated with IPMN pathology

Radiomic Feature	Category	Odds Ratio	Lower 95% CI	Upper 95% CI	AUC (95% CI)	*P*-value
Fourier Descriptor Layer 1	Texture	0.42	0.18	0.97	0.69 (0.51-0.87)	0.043
Histogram Energy Layer 1	Texture: Histogram	0.18	0.05	0.73	0.79 (0.64-0.94)	0.017
Histogram Entropy Layer 1	Texture: Histogram	3.77	1.34	10.6	0.77 (0.62-0.93)	0.012
Co-occurrence matrix features OF1 G1 CONTRAST Layer 1	Texture: Co-occurrence/ Run-length	8.08	1.40	46.7	0.79 (0.64-0.94)	0.020
Run-length features G1 D0 HGRE Layer 1	Texture: Co-occurrence/ Run-length	4.30	1.37	13.5	0.79 (0.63-0.95)	0.013
Run-length features G1 D0 LGRE Layer 1	Texture: Co-occurrence/ Run-length	0.11	0.01	0.88	0.79 (0.64-0.94)	0.038
Laws features E5 E5 Energy Layer 1	Texture: Laws	0.06	0.01	0.65	0.74 (0.58-0.91)	0.020
Laws features L5 S5 Energy Layer 1	Texture: Laws	0.21	0.05	0.91	0.73 (0.56-0.89)	0.037
Laws features R5 E5 Energy Layer 1	Texture: Laws	0.20	0.05	0.91	0.71 (0.53-0.89)	0.038
Wavelet decomposition. P1 L3 C1 Layer 1	Texture: Wavelet	2.80	1.07	7.34	0.74 (0.57-0.91)	0.036
Wavelet decomposition. P1 L3 C2 Layer 1	Texture: Wavelet	2.69	1.02	7.12	0.75 (0.59-0.92)	0.046
Border length (Pxl)	Non-texture: Size & Shape	2.61	1.08	6.31	0.74 (0.57-0.92)	0.033
Width (Pxl)	Non-texture: Size & Shape	2.76	1.20	6.32	0.77 (0.61-0.93)	0.017
Radius of largest enclosed ellipse	Non-texture: Size & Shape	0.44	0.19	0.99	0.78 (0.61-0.94)	0.048

### Integration of radiomic data with other data types

**Figure 3:**
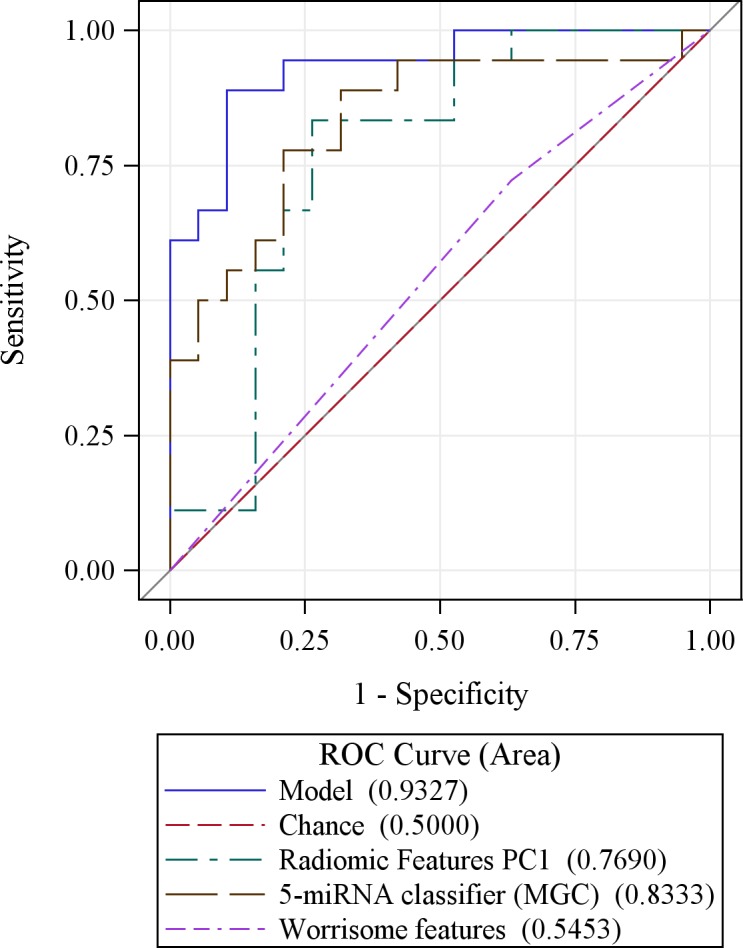
An integrative model combining radiomic features (PC1) with the 5-miRNA genomic classifier (MGC) is more accurate at predicting malignant IPMN pathology than either variable alone and is substantially more accurate for prediction than worrisome radiologic features A final model combining worrisome features, radiomic features, and the MGC has potential to have high accuracy, with an AUC value approximating 0.93.

A model that combined radiomic features and the MGC had an AUC = 0.92 and estimates of sensitivity (83%), specificity (89%), PPV (88%), and NPV (85%) that were superior to models not based on these data types that relied on demographic or standard imaging features alone, particularly worrisome features (Table 3). When integrating standard worrisome radiologic features with radiomic features and the MGC, the diagnostic performance of the model increased further (AUC = 0.93 (95% CI: 0.85-1.00) (Figure 3) than the models based on worrisome features alone (p < 0.001) and radiomic PC1 alone (p = 0.037), with enhanced estimates of sensitivity, PPV, and NPV, each at 89% (Table 4). As expected, models that considered presence of high-risk stigmata in conjunction with radiomic data performed well.

**Table 3:** Diagnostic performance of preliminary models to predict malignant IPMN pathology in the study cohort

Modela	Variables included	AUC (95% CI)	Sensitivity	Specificity	Positive predictive value	Negative predictive value
Demographic and clinical data	Age at diagnosis, gender, presence of symptoms	0.73 (0.56-0.89)	0.83	0.55	0.63	0.79
Standard imaging data	High risk stigmata	0.84 (0.72-0.96)	0.83	0.85	0.83	0.85
Genomic data	5-miRNA genomic classifier (MGC)	0.83 (0.69-0.97)	0.78	0.80	0.78	0.80
Standard imaging + genomic data	High risk stigmata, MGC	0.95 (0.88-1.00)	0.94	0.90	0.89	0.95
Standard imaging data	Worrisome features	0.54 (0.38-0.69)	0.72	0.35	0.50	0.58
Standard imaging + genomic data	Worrisome features, MGC	0.83 (0.69-0.97)	0.83	0.80	0.79	0.84
Radiomic data	Radiomic PC1 classifier	0.77 (0.61-0.93)	0.83	0.74	0.75	0.82
Radiomic + genomic data	Radiomic PC1 classifier + MGC	0.92 (0.83-1.00)	0.83	0.89	0.88	0.85
Standard imaging+ radiomic+ genomic data	Worrisome features, Radiomic PC1 classifier + MGC	0.93 (0.85-1.00)	0.89	0.89	0.89	0.89

^a^ Full models include 20 benign and 18 malignant IPMNs.

### Cross-validation and correlative analysis

Evaluation of uncertainty by 10-fold cross-validation showed robust estimates of diagnostic performance with AUC above 0.75 for most models (Supplementary Table 2). Specifically, a model that combined radiomic features with the MGC had an AUC = 0.87 (95% CI: 0.84-0.89) and was more accurate than demographic characteristics and worrisome features at predicting malignant pathology. Finally, preliminary analyses revealed that the radiomic ‘features PC1’ was associated with high risk stigmata (p = 0.0009) and worrisome features (p = 0.0006), but not with the MGC (p > 0.05).

## DISCUSSION

Due to their malignant potential, the identification of an IPMN generates anxiety, the need for subsequent imaging, possible invasive testing or surgery, and huge economic costs (54). Thus, the value of developing a noninvasive, cost-effective approach to accurately distinguish malignant from benign IPMNs cannot be overstated because it would enable individuals with malignant lesions to undergo lifesaving surgery while sparing those with benign lesions the inconvenience, morbidity, and cost of major surgery. Here we conducted the first proof-of-concept radiogenomic study to noninvasively evaluate the clinical utility of radiomic features as predictors of malignant IPMNs alone and in combination with a blood-based miRNA genomic classifier discovered by our team [14]. Consistent with data from other cancers which suggest that complementary biomarkers will increase specificity of standard-of-care images [40, 41], our preliminary data infer that incorporating radiomic and miRNA expression data from images and blood obtained through standard of care has potential to accelerate discovery of a noninvasive multimodal approach to rapidly provide clinically-actionable information to improve pre-operative prediction of IPMN malignancy, especially for patients who do not present with high-risk stigmata. Such an approach would also minimize the potential for sampling bias and risks associated with tissue biopsy-dependent approaches that do not capture tumor heterogeneity.

Radiomics provides a noninvasive, fast, low cost, and repeatable way of investigating quantitative radiophenotypes that may potentially personalize care for patients with pancreatic cancer precursors. After applying a semiautomatic segmentation process that minimizes operator interaction and has been shown to provide accurate and reproducible boundaries [19, 21, 42], we successfully extracted 112 preoperative CT radiomic features, and revealed 14 features that differentiated malignant from benign IPMNs (p < 0.05). Of the 14 features, 11 were textural (histogram, wavelets, laws, or run-length/co-occurrence) and 3 were non-textural and from the size and shape category. Texture of CT scans to a clinical radiologist is usually attributed to gray-level changes seen by the expert whereas texture in traditional image processing refers to the spatial arrangement of color or intensity in a localized region or whole scene. It is of interest that in this and other radiomic investigations [43], the most important characteristics to separate the two clinical outcomes were textural based on run length and co-occurrence (with few others). Interestingly, Hanania et al (submitted, Oncotarget) recently conducted a study in which they describe the use of quantitative radiomic features for risk stratification of IPMNs. Hanania et al also identified fourteen top-performing radiomic features (all textural within the gray-level co-occurrence matrix) as differentiating between benign and malignant pathology. Using a cross-validated design, the top-performing logistic regression model yielded an AUC = 0.96, with a sensitivity and specificity of 97% and 88%, respectively. Because the study by Hanania et al does not have matched plasma miRNA data and our groups used different methodology for acquisition, segmentation, feature extraction, and analysis, the opportunity for meaningful independent validation of each other's findings is not possible at this point in time. However, our teams plan to work together to prospectively evaluate this topic area and develop standard operating procedures though mechanisms such as the Molecular and Cellular Characterization of Screen-Detected Lesions (MCL) U01 consortium, supported by NCI's Divisions of Cancer Prevention and Cancer Biology.

In the current study, when the 14 textural and non-textural features were combined via PCA to generate a feature PC1 score that explained 61% of the variability in the data, the radiomic features had a diagnostic accuracy for predicting malignant pathology that was higher than standard worrisome radiologic features or demographic and clinical data elements, especially when combined with the blood-based MGC (Table 3). Indeed, blood-based biomarkers have potential to reduce the false positive and overdiagnosis rates of CT scans [44]. To date, no blood-based biomarker has proven useful in clinical practice for the early detection of PC [45]. miRNAs represent ideal candidates for overcoming limitations of single blood-based biomarkers because they can reflect physiological and pathological conditions and act as extracellular messengers of biological signals derived from the cross talk between the tumor and its microenvironment [46]. Although study limitations include potential model overfitting due to the small sample size and the need for further multivariable modeling, we did perform cross-validation analyses which indicated robustness of the model using the combination of a MGC with radiomic features from preoperative CT scans. The results suggest these data types may significantly improve the ability to noninvasively risk stratify IPMNs for resection or surveillance. Additionally, external validation in an independent data set is warranted; plans are underway to do this as part of a multi-center prospective study since MGC data is not available for additional retrospective cohort participants.

We recognize that the retrospective design of the current investigation is vulnerable to selection bias since the main inclusion criteria was having a pathologic diagnosis obtained through surgical resection. However, a key advantage of this retrospective series is the ability to integrate several important data types in a relatively large sample size compared to other recent radiomic studies. Another challenge is that CT imaging protocols and scanners, acquisition procedures, slice selection and thickness, and reconstruction parameters may change over time and be coupled with confounding factors stemming from imaging variability and heterogeneity within and between patient cohorts. We acknowledge that inconsistencies and heterogeneity in scans derived from standard diagnostic procedures may play a role in the derived inference and that improvements in diagnostic performance attributed to radiomic features may be minimal in this small dataset. Furthermore, specific to IPMNs, it may be necessary to separate segmentation of nodular and cystic components in future investigations. In our experience, only a subset of IPMN cases had a separate soft tissue component that could be clearly delineated from cystic components; this is an area we plan to evaluate further in the future. Nevertheless, we expect the contribution of radiomic features in the prediction models will be greater once CT acquisition procedures are harmonized. In previous analyses of lung lesions and other cancers [21, 27], we evaluated reproducibility of CT-based image features subjected to typical patient level variability; known stable features were used to show the translational potential for multi-institutional application of radiomics [27]. To ensure robust decision support for patients with IPMNs, a radiomics-based classifier will require inclusion of informative, non-redundant features that have high reducibility and stability and are scanner independent. Moreover, engineers and domain expertise are needed to validate models across platforms, potentially using deep learning approaches such as neural net pathway analyses. Furthermore, as part of the National Cancer Institute (NCI) Quantitative Imaging Network (QIN) (http://imaging.cancer.gov/informatics/qin), we plan to work with other leaders in the field to assess variability of radiomic metrics across institutions due to system and reader inputs (segmentation, seeding, etc.).

In summary, this proof of concept study represents the first we are aware of to integrate clinical factors, radiomic features, and blood-based miRNA expression data [14] into a statistical model that could potentially provide a robust and noninvasive predictor of malignant IPMN pathology. Our preliminary data and that of Hanania et al (submitted, Oncotarget) suggest a radiomic CT approach could have a previously unappreciated impact, value, and practicality in capturing readily available information not currently analyzed in CT imaging studies to aid in managing pancreatic cysts, a goal in line with QIN initiatives. Larger studies are needed to prospectively explore the diagnostic performance of textural and non-textural radiomic CT features (and possibly features extracted from other modalities such as MRIs or PET/CTs) and the blood-based miRNA classifier as noninvasive predictors of malignant IPMN pathology. If proven useful, such a multimodal approach may lead to a reduction in pancreatic resections and missed opportunities for cures.

## MATERIALS AND METHODS

### Study population and data

A prospectively maintained clinical database was retrospectively reviewed to identify individuals who underwent a pancreatic resection for an IPMN between 2006 and 2011 at Moffitt Cancer Center and Research Institute (Moffitt) and had provided written consent for blood, imaging, and clinical data to be donated pre-operatively for research through protocols approved by the Institutional Review Board (IRB) of the University of South Florida, including Total Cancer Care [47]. IRB approval was granted for the research described herein (IRB#Pro4971). For all cases, demographic and clinical data (presenting systems, age at diagnosis, past medical and surgical history, current and past medication use, and information on a uniform set of known and suspected cancer risk factors such as smoking and alcohol history, family history, and body mass index) was obtained from the electronic medical record and patient questionnaire. Detailed imaging studies, surgical details, pathology results, lab values (serum CA-19-9, bilirubin, albumin), and treatment information was collected from the medical record and Moffitt's Cancer Registry.

### Histopathologic analysis

Pathologists with expertise in PDAC and IPMN pathology (KJ, DC, BAC) used hematoxylin and eosin (H&E) stained slides from selected blocks to histologically confirm the diagnosis and degree of dysplasia using World Health Organization guidelines [48]. The final diagnosis represented the most severe grade of dysplasia observed in the neoplastic epithelium. None of the cases received pre-operative chemotherapy or radiation. ‘Malignant’ cases were defined by high-grade dysplasia or invasive carcinoma and ‘benign’ cases were defined by low- or moderate-grade dysplasia.

### miRNA expression data

Preoperative plasma miRNA expression data was previously generated [14] for 42 surgically-resected, pathologically-confirmed IPMN cases (21 malignant and 21 benign). Briefly, one 0.5-mL cryovial of plasma was retrieved and thawed for each identified case. To control for variance in starting material and efficiency of RNA extraction, RNA spike-in miRNAs (synthetic control templates) were used. Total RNA isolation was performed on 500 uL of plasma using the Plasma/Serum Circulating and Exosomal RNA Purification Mini Kit (Slurry Format) from Norgen Biotek (Ontario, Canada), and total RNA concentration and integrity was assessed using the NanoDrop spectrophotometer (NanoDrop Technologies, Waltham, MA) and an Agilent Bioanalyzer (Agilent, Santa Clara, CA). Since hemolysis can confound studies of plasma miRNAs, the possibility of hemolysis was assessed.

The nCounter™ Human v2 miRNA Expression Assay Codeset (Nanostring Technologies, Seattle, WA, USA) was used to quantify the abundance of a pre-defined panel of 800 human miRNAs and built-in controls. Raw miRNA counts underwent technical and biological normalization and log2-transformation. The most deregulated miRNAs that differed between the benign and malignant groups were identified using the linear models for microarray data (LIMMA) method and a principal component analysis (PCA) approach (14). Since miRNAs can be over- or under-expressed, we used PCA to combine the most deregulated miRNAs and generate an overall ‘IPMN-risk score’ based on the first principal component (PC1). A focused analysis of the 42 IPMN cases showed that five miRNAs (miR-200a-3p, miR-1185-5p, miR-33a-5p, miR-574-3p, and miR-663b), a miRNA genomic classifier (‘MGC’), discriminated between malignant and benign IPMNs (p < 0.05). The overall expression of PC1 was lower in malignant compared to benign IPMNs (p = 0.005), was significantly associated with malignant status (OR (95% CI): 0.36 (0.16, 0.83), p = 0.017), and had an AUC value of 0.73 (95% CI: 0.58-0.89) in discriminating between groups.

### CT imaging

The majority of CT scans from this series of patients were obtained on the Siemens Sensation (16, 40, or 64) using a CT angio (CTA) pancreas protocol (Supplementary Table 1). Our standard operating procedure includes obtaining 3 mm axial CT slice images of the abdomen from the superior liver capsule to the iliac crests without contrast. Optiray-370 contrast is then injected intravenously at a rate of 3.5 ml/sec. The volume of Optiray-370 contrast injected follows a weight-based scale. Arterial phase imaging is triggered by contrast bolus tracking of the abdominal aorta at a Hounsfield Unit of 100-120. Arterial phase images of the abdomen are obtained ∼20 seconds after contrast injection, and venous phase images of the abdomen are obtained ∼60 seconds after injection. 3 mm coronal reconstruction images (B30/B31f) of the abdomen are also performed for each phase (noncontrast, arterial phase, venous phase).

### CT acquisition and feature selection and extraction

Preoperative CT images were available for 38 of the 42 surgically-resected, pathologically-confirmed IPMN cases with available matched preoperative miRNA expression data generated in our previous publication [14]. Thus, overlap exists for 38 of the study participants in the current report and our previous publication [14]; the prior article [14] dealt with the development of a miRNA classifier whereas in this manuscript we emphasize standard radiologic features and radiomic features as predictors of IPMN pathology with and without the miRNA classifier. CT images were obtained from Moffitt's GE Centricity Picture Archiving and Communication System (PACS). Most cases underwent contrast-enhanced CTs within 3 months prior to surgery. Our lead radiologist who has over 5 years of experience reviewing IPMN cases (J.C.) and the entire analytic team were blinded to the pathological diagnosis. CT images were reviewed for standard radiologic features encompassing ‘high-risk stigmata’ and ‘worrisome features’ represented in consensus guidelines [5]. The scan reconstruction and central slice was selected by J.C. Axial venous phase images (3 mm) were used for most patients because of the tumor/background contrast. Arterial phase or coronal images were used as needed. In the event multifocal disease was present, we characterized the most concerning cyst that corresponded to one that was ultimately resected. J.C. identified the region of interest (ROI) by helping to outline the peripheral margin of tumors in their entirety, capturing both solid (nodular) and cystic components. The radiomics team then marked the ROI using Definiens /GE AWS Advanced Visualization software. Based on previous successes [19, 21], the entire tumors were identified (solid and cystic components) using a semi-manual version of a single click semi-automated ensemble segmentation algorithm within the Definiens Developer XD (Munich, Germany) software platform. Target lesions were segmented, with a second radiologist (Q.L, Resident Radiologist with over 2 years of experience) finalizing the segmentation boundaries on the CT slices. This approach eliminates the need for a manually drawn boundary while providing robust, reproducible, and consistent delineation of the tumor region across CT slices [19, 21]. The segmentation algorithm has also been shown to reduce inter-observer variability while capturing intricacies of the tumor boundary [42].

We then extracted categories of non-texture and texture features. Non-texture features measure tumor size (volume, diameter, border length), shape (compactness, asymmetry), and location, whereas texture features measure properties such as smoothness, coarseness, and regularity. We focused on evaluating two-dimensional (2D) quantitative features in the middle CT slice. To minimize analysis problems inherent with testing hundreds of features in a limited sample size, we reduced dimensionality by limiting the number of imaging features to 112. Of the 112 features, 18 were non-textural and 94 were textural (10 histogram in hounsfield units, 27 co-occurrence/run-length, and 57 laws and wavelets). In house algorithms for feature extraction and quantification of segmented regions were implemented by custom routines in the Definiens Platform.

### Statistical analyses

For a selected set of variables, descriptive statistics were calculated using frequencies and percents for categorical variables and means and standard deviations (SD) for continuous variables.

After feature extraction, the Pearson correlation coefficient was used to identify and filter out 55 highly correlated (or redundant) radiomic features, leaving 57 features for analysis. Simple logistic regression models were used to explore associations with IPMN pathology. Principal component analysis (PCA) was also performed to reduce radiomic feature data dimensionality and to generate an index score defined by the first principal component (PC1); PC1 was evaluated for its association with malignant status using logistic regression. The sensitivity, specificity, positive predictive value (PPV), negative predictive value (NPV), and accuracy of the new radiomic features were calculated by estimating the optimal cutpoint using Youden's index [49], with pathological diagnosis as the gold standard. Estimates of diagnostic performance generated from radiomic models were generated with and without the MGC data and were compared to those obtained when evaluating standard radiologic and clinical features [5]. DeLong's test was used to compare the area under correlated ROC curves [50]. To evaluate model performance, repeated (10,000 times) 10-fold cross validation was performed. The average and 95% confidence intervals of accuracy, sensitivity, specificity, PPV and NPV were estimated. In each 10-fold cross-validation, data were split into 10 subsets. By holding one subset of data (test set), the remaining 9 subsets were used as a training set to build a model for prediction evaluation in the test set. The process continued until each subset was used as the test set. By testing the model on a test set (not used in estimation), the cross-validation approach aimed to reduce over-fitting. Although prediction on the test set (treated as new data) would likely increase uncertainty and therefore reduce performance, it provides a great tool to evaluate robustness of the model. Finally, we evaluated if the most statistically significant imaging features were correlated with one another or the MGC using two-sample t-tests or Wilcoxon rank sum tests.

## SUPPLEMENTARY MATERIALS TABLES


