# Supplementary material for: Combining radiomic features with a miRNA classifier may improve prediction of malignant pathology for pancreatic intraductal papillary mucinous neoplasms
Source: Oncotarget. 2016 Aug 31;7(52):85785–97. doi: 10.18632/oncotarget.11768 (PMC5349874; doi:10.18632/oncotarget.11768)
Supplement: Supplementary file 1 [file oncotarget-07-85785-s001.pdf]

Combining radiomic features with a miRNA classifier may improve prediction of malignant pathology for pancreatic intraductal papillary mucinous neoplasms

Supplementary Material

Supplementary Table 1: CT scanner parameters.

Manufacturer	Model	Count	Convolutional Kernel	Tube Current (ma)	Software	Institutions
Seimens	Sensation 16 (22)	34	B30f (15)	188 - 401	Syngo-CT2006G (11), VA70C (6), VB10B(5)	Moffitt Cancer Center (32), Consortium Centers (2)
	Sensation 40 (6)		B31f (15)	153-520	Syngo-CT2007S	
	Sensation 64 (3)		B31S (3)	136-169	Syngo-CT2007S	
	Volume zoom (3)		B40f (1)	107	VA47C	
Toshiba	Acquilion	2	FC11 (2)	300	V1.41ER001	Consortium Centers (2)
GE	Light Speed 16	1	Standard (1)	340	LightSpeedApps405I.2_H4.0M5	Consortium Centers (1)

Supplementary

Table 2. Diagnostic performance of preliminary models to predict malignant IPMN pathology in the study cohort based on 10-fold cross validation

Variables	AUC	Accuracy	Sensitivity	Specificity	PPV	NPV
**Age at diagnosis, gender, presence of symptoms**	0.6 (0.55-0.64)	0.57 (0.5-0.66)	0.6 (0.44-0.72)	0.54 (0.45-0.65)	0.54 (0.47-0.62)	0.6 (0.52-0.69)
**High risk stigmata**	0.74 (0.72-0.76)	0.84 (0.84-0.84)	0.83 (0.83-0.83)	0.85 (0.85-0.85)	0.83 (0.83-0.83)	0.85 (0.85-0.85)
**MGC**	0.79 (0.75-0.82)	0.72 (0.68-0.76)	0.78 (0.78-0.78)	0.66 (0.6-0.75)	0.67 (0.64-0.74)	0.77 (0.75-0.79)
**High risk stigmata, MGC**	0.87 (0.82-0.89)	0.85 (0.79-0.89)	0.88 (0.78-0.94)	0.81 (0.75-0.9)	0.81 (0.76-0.89)	0.89 (0.8-0.94)
**Worrisome features**	0.38 (0.26-0.47)	0.47 (0.32-0.53)	0.66 (0.5-0.72)	0.29 (0.15-0.35)	0.46 (0.35-0.5)	0.49 (0.25-0.58)
**Worrisome features, MGC**	0.77 (0.72-0.8)	0.75 (0.71-0.79)	0.76 (0.67-0.83)	0.74 (0.65-0.8)	0.73 (0.67-0.78)	0.77 (0.71-0.83)
**Radiomic PC1**	0.75 (0.73-0.77)	0.76 (0.76-0.78)	0.83 (0.83-0.83)	0.69 (0.68-0.74)	0.72 (0.71-0.75)	0.81 (0.81-0.82)
**Radiomic PC1, MGC**	0.87 (0.84-0.89)	0.83 (0.78-0.86)	0.77 (0.67-0.83)	0.88 (0.79-0.89)	0.86 (0.78-0.88)	0.8 (0.74-0.85)
**Worrisome features, Radiomic PC1, MGC**	0.85 (0.81-0.88)	0.81 (0.76-0.86)	0.83 (0.78-0.89)	0.79 (0.68-0.89)	0.79 (0.71-0.88)	0.83 (0.78-0.89)

Abbreviations: AUC=Area underneath the curve; PPV=positive predictive value; NPV=negative predictive value; MGC=miRNA genomic classifier; PC1=principal component 1
